# Integrating Nutrition Interventions into an Existing Maternal, Neonatal, and Child Health Program Increased Maternal Dietary Diversity, Micronutrient Intake, and Exclusive Breastfeeding Practices in Bangladesh: Results of a Cluster-Randomized Program Evaluation

**DOI:** 10.3945/jn.117.257303

**Published:** 2017-10-11

**Authors:** Phuong Hong Nguyen, Sunny S Kim, Tina Sanghvi, Zeba Mahmud, Lan Mai Tran, Sadia Shabnam, Bachera Aktar, Raisul Haque, Kaosar Afsana, Edward A Frongillo, Marie T Ruel, Purnima Menon

**Affiliations:** 1 Poverty, Health and Nutrition Division, International Food Policy Research Institute (IFPRI), Washington, DC;; 2 FHI 360, Washington, DC;; 3 BRAC, Dhaka, Bangladesh; and; 4 Department of Health Promotion, Education, and Behavior, University of South Carolina, Columbia, SC

**Keywords:** Bangladesh, breastfeeding, cluster-randomized trial, community mobilization, dietary diversity, interpersonal counseling, maternal undernutrition, micronutrient intake

## Abstract

**Background:** Maternal undernutrition is a major concern globally, contributing to poor birth outcomes. Limited evidence exists on delivering multiple interventions for maternal nutrition simultaneously. Alive & Thrive addressed this gap by integrating nutrition-focused interpersonal counseling, community mobilization, distribution of free micronutrient supplements, and weight-gain monitoring through an existing Maternal, Neonatal, and Child Health (MNCH) program in Bangladesh.

**Objectives:** We evaluated the effect of providing nutrition-focused MNCH compared with standard MNCH (antenatal care with standard nutrition counseling) on coverage of nutrition interventions, maternal dietary diversity, micronutrient supplement intake, and early breastfeeding practices.

**Methods:** We used a cluster-randomized design with cross-sectional surveys at baseline (2015) and endline (2016) (*n* ∼ 300 and 1000 pregnant or recently delivered women, respectively, per survey round). We derived difference-in-difference effect estimates, adjusted for geographic clustering and infant age and sex.

**Results:** Coverage of interpersonal counseling was high; >90% of women in the nutrition-focused MNCH group were visited at home by health workers for maternal nutrition and breastfeeding counseling. The coverage of community mobilization activities was ∼50%. Improvements were significantly greater in the nutrition-focused MNCH group than in the standard MNCH group for consumption of iron and folic acid [effect: 9.8 percentage points (pp); 46 tablets] and calcium supplements (effect: 12.8 pp; 50 tablets). Significant impacts were observed for the number of food groups consumed (effect: 1.6 food groups), percentage of women who consumed ≥5 food groups/d (effect: 30.0 pp), and daily intakes of several micronutrients. A significant impact was also observed for exclusive breastfeeding (EBF; effect: 31 pp) but not for early initiation of breastfeeding.

**Conclusions:** Addressing nutrition during pregnancy by delivering interpersonal counseling and community mobilization, providing free supplements, and ensuring weight-gain monitoring through an existing MNCH program improved maternal dietary diversity, micronutrient supplement consumption, and EBF practices. This trial was registered at clinicaltrials.gov as NCT02745249.

## Introduction

Maternal undernutrition is a major public health concern globally, contributing to poor fetal and early childhood growth and increased infant morbidity and mortality, with long-term adverse consequences for child development and life-long health (1). There is growing recognition of the importance of maternal nutrition interventions as part of antenatal care (ANC) to improve maternal and infant health outcomes (2). In 2016, the WHO issued new guidelines on ANC for a positive pregnancy experience (3), with high priority given to nutrition through dietary interventions and micronutrient supplementation, together with health system interventions to improve the use and quality of ANC. The successful implementation of these interventions will contribute to achieving the global nutrition targets for 2025 (4).

Reviews of the interventions used to improve maternal nutrition during pregnancy and their effects on maternal and infant health noted that the overall quality of the evidence was low to very low due to heterogeneity in the studies, poor study designs, and lack of control for potential confounding (5, 6). A review of 5 antenatal nutrition education trials (all in high-income countries except for 1 in Bangladesh) showed increased protein intake (7 g/d; 95% CI: 3, 11 g/d), reduced risk of preterm birth (RR: 0.46; 95% CI: 0.21, 0.98) and low birth weight (RR: 0.04; 95% CI: 0.01, 0.14), and increased birth weight among newborns delivered by undernourished [BMI (in kg/m^2^) <18.5] women (490 g; 95% CI: 428, 552 g) (6). Balanced energy and protein supplementation (12 trials; 7 in low- and middle-income countries, 6705 women) reduced the risk of still birth (by 40%) and small-for-gestational age (by 21%) and increased birth weight (by 41 g). A meta-analysis of 34 studies (11 of which were in low- and middle-income countries) showed that maternal nutrition education and counseling significantly improved gestational weight gain (by 0.45 kg) and birth weight (by 105 g) and reduced the risk of maternal anemia in late pregnancy (by 30%) and of preterm delivery (by 19%) (5). The impacts of micronutrient supplements, such as iron and folic acid (IFA) (7), calcium (8), vitamin A (9), and zinc (10), on maternal and child health outcomes are well documented. Most micronutrient-focused studies, however, reported findings from single interventions.

Limited evidence exists on the benefits of delivering multiple interventions for maternal nutrition. Studies in India (11, 12), Nepal (13), and Senegal (14) showed higher effects when combining nutrition education and IFA supplementation than with nutrition education or supplementation alone. The meta-analysis of 34 studies showed that the impact of nutrition education and counseling was greater when combined with direct nutrition support such as food, micronutrient supplements, or nutrition safety nets (5).

The Alive & Thrive initiative addressed these gaps through integrating multiple nutrition interventions into an existing Maternal, Neonatal, and Child Health (MNCH) program in Bangladesh, a country with a high prevalence of maternal and child undernutrition (15), which we refer to as “nutrition-focused MNCH.” This article reports findings from a cluster-randomized impact evaluation comparing the nutrition-focused MNCH with a standard MNCH program on *1*) coverage and use of maternal nutrition interventions, *2*) consumption of diversified foods and adequate amounts of macro- and micronutrients during pregnancy, and *3*) early breastfeeding practices.

## Methods

### 

#### Study context and intervention description.

BRAC, a large national nongovernmental organization in Bangladesh, has been providing community-based MNCH services, including standard nutrition interventions, as part of antenatal health care since 2010 (16, 17). The MNCH program operates in both rural (14 districts) and urban (11 city corporations) areas. Services provided by the standard MNCH program included family planning, identification of pregnancies, ANC, delivery and postnatal care, essential neonatal care, management of neonatal and childhood illnesses, vaccination, and referral for complications. In 2015, Alive & Thrive designed an intensified, nutrition-focused package of interventions to include in the existing MNCH program with the goal of improving maternal diet quality, micronutrient intakes, and breastfeeding practices. Although some nutrition interventions were provided in the standard MNCH program (**Table 1**), the nutrition-focused MNCH included greater specificity of interpersonal counseling, provided free supplements, conducted weight-gain monitoring during pregnancy, engaged fathers more explicitly, and included community mobilization activities.

**TABLE 1:** Differences between intervention and comparison areas^1^

Interventions	Standard MNCH areas (MNCH alone)	Nutrition-focused MNCH areas (MNCH + nutrition interventions)
Counseling on diet diversity and quantity	Standard nutrition education messages during ANC contacts	More intensified and greater specificity of interpersonal counseling
	Health worker’s tasks:	Health worker’s tasks:
	- Counseled the pregnant woman on overall diet, no specific messages, no counseling for family members	- Counseled the pregnant woman, husband, and family members about importance of maternal nutrition, benefits of consuming balanced diet, consequences of poor nutrition for maternal, fetal, and child health, and causes of poor nutrition during pregnancy
	- Did not have food demonstrations	- Demonstration of the preparation of a low-cost balanced diet with locally available nutritious foods (type, quantity, frequency, diversity); health workers demonstrated with the foods available at the pregnant woman’s home during an ANC visit; health workers showed daily quantities of food with a 250-mL bowl
		- If food from any food group was missing during demonstration, then health workers counseled the mother about missing nutrients and motivated her and family to provide foods from all groups
	Health volunteer’s tasks:	Health volunteer’s tasks:
	- Provided general messages on adequate maternal nutrition; no food demonstrations, no specifics on identifying local foods	- Accompanied health workers during home visit and assisted them in demonstrating how to prepare a recommended balanced diet
		- In the subsequent month, health volunteers would follow up with the counseled pregnant women and reinforce the messages
		- Helped the family to identify low-cost, locally available diverse nutritious foods, seasonal vegetables, and fruit
IFA and calcium supplements	Sale of IFA and calcium tablets by health volunteer	Free IFA and calcium tablets provided by health workers with an emphasis on compliance during home visits
	Free distribution of IFA at government health facilities	Counseled women on benefits of IFA and calcium, consequences of IFA or calcium deficiency, doses and duration of IFA and calcium that should be followed, side effects and ways to minimize them, and foods that inhibit their absorption
	Counseled women to take IFA but did not have specific messages on various topics	Requested women to preserve empty strips and count them in the subsequent visit
	Did not monitor if women took tablets or how many women took	
Weight measurement	No specific activities on weight measurement and weight-gain monitoring	Measured monthly pregnancy weight gain, filled in the chart, and drew a line corresponding to weight gain
		Counseled pregnant women on adequate weight gain
Taking rest and avoiding heavy workload	General message on taking rest	Counseled pregnant women to take 2 h of rest in the daytime and to avoid heavy workloads
		Encouraged the family to share the mother’s work
Counseling on breastfeeding	Standard messages in third trimester	Core counseling package
		Frontline workers provided with refresher training on the topic every month
		More frequent counseling
		More frequent reinforcing messages by frontline workers
		Provided support and problem-solving for any issues that occurred
Community mobilization	No community mobilization activities	Husbands’ forums held at second and third trimesters to motivate husbands for taking special care of their wife and to educate husband on the benefits of proper diet and IFA and calcium; husbands were encouraged to ensure adequate supplies of IFA, calcium, and foods in the house and to remind their wives to follow the recommended practices
		Community interactive media events on special topics such as ensuring nutrition and care for pregnant women, 5 rules for pregnant women (diet quality, quantity, IFA, calcium, and weight gain), where to find nutritious food, initiation of breastfeeding right after birth, and breast milk is enough ≤6 mo of age
Number of home visits	Health workers and health volunteers: monthly visits from identification of pregnancy to delivery	Health workers: 7 visits during pregnancy, 5 visits during postpartum period
		Health volunteers: 14 visits during pregnancy, 10 visits during postpartum period
Incentive structure	Standard incentive indicators:	Standard incentives with 4 additional indicators:
	- Identification of pregnancy	- Health volunteers conducted home visits 2 times/mo
	- Early initiation of breastfeeding	- ≥5 groups of food consumed by pregnant women
		- 30 IFA/30 calcium tablets consumed by pregnant women
		- Health volunteers assisted health workers in measuring weights of all pregnant women

1 ANC, antenatal care; IFA, iron and folic acid; MNCH, Maternal, Neonatal, and Child Health.

In both nutrition-focused MNCH and standard MNCH models, interpersonal counseling was delivered by 2 types of frontline workers, a salaried health worker (Shasthya Kormi) and a community health volunteer (Shasthya Shebika). In the nutrition-focused MNCH model, the health worker conducted monthly home visits and one-on-one ANC sessions for all pregnant women to deliver the following interventions: *1*) demonstration of a specific diet plan (both quality and quantity), *2*) provision of free supplements [IFA (60 mg elemental Fe and 400 μg folic acid) and calcium (500 mg) tablets] and advising on their use, *3*) measurement of weight and explaining weight-gain patterns, *4*) counseling on resting, and *5*) engaging other family members to ensure enough foods and supplements and support for the pregnant women.

During the postpartum period, health workers counseled mothers on a specific diet plan during lactation and promoted optimal breastfeeding practices. Health workers were tasked with conducting 7 visits during pregnancy and 5 visits during the postpartum period. Health volunteers conducted 2 visits per household per month and provided follow-up messages to reinforce the demonstrations and counseling given by health workers. In the standard MNCH model, visits were less frequent with much less focus on nutrition.

In the nutrition-focused MNCH model, health workers and volunteers were highly trained and closely supervised, and health volunteers received performance-based cash incentives on the basis of each new mother reached, home visits, and mothers practicing the recommended behaviors. Cash incentives were limited in the standard MNCH model (Table 1). In the nutrition-focused MNCH program, regular monitoring and supervision by BRAC staff, district managers, headquarters staff, and an independent team of 5 monitors were provided to track the performance of frontline workers (through direct observation) and practices of mothers (through interview and observation). Each monitor visited ∼70 randomly selected households each month.

Community mobilization in the nutrition-focused MNCH model involved husbands’ forums and video shows. Husbands of pregnant women were reached twice during pregnancy (in the second and third trimesters) through forums to discuss several topics related to ensuring adequate supplies of foods and micronutrient supplements, and supporting their wives to practice optimal nutrition behaviors. Video shows and interactive communication were carried out in the community (3 videos focused on nutrition during pregnancy and 2 focused on breastfeeding practices), targeting women, their husbands and family members, local leaders, village doctors, and government health workers. There was no community mobilization in the standard MNCH program. The maternal nutrition interventions were implemented from August 2015 to December 2016. Coverage of the interventions was not affected by the rainy season, because the monitoring data showed that the coverage was similar across different months of the year.

#### Evaluation design.

A cluster-randomized, nonblinded design was used for evaluation. Twenty subdistricts (*upazilas*) from 4 districts (Mymensingh, Rangpur, Kurigram, and Lalmonirhat) in which BRAC’s rural MNCH program existed were randomly selected and randomly assigned to nutrition-focused MNCH or standard MNCH areas. Cross-sectional household surveys were conducted at baseline (2015) and at endline (2016) in the same villages at the same time of year (June–August). This design, which was longitudinal at the village level, was optimal for assessing whether the nutrition-focused MNCH resulted in population shifts in coverage and use of the interventions and changes in behaviors compared with the standard MNCH.

#### Sample size estimations.

There were 2 samples: *1*) recently delivered women with children <6 mo of age, which provided the best opportunity to assess most of the outcome indicators, and *2*) pregnant women in the second and third trimesters of pregnancy, which allowed for the assessment of dietary diversity during pregnancy. Sample sizes were estimated on the basis of baseline prevalence of primary outcomes [i.e., IFA consumption, dietary diversity, and exclusive breastfeeding (EBF)], expected change after intervention, power to detect those differences at 0.80, level of significance at 0.05, and intraclass correlation of 0.03 estimated from our previous data for EBF in Bangladesh (18). Assuming a baseline IFA consumption of 95 tablets on the basis of the 2014 Bangladesh Demographic and Health Survey (19), a total sample of 2000 recently delivered women (1000/group) would have ≥80% power to detect a difference of 20 IFA tablets between study groups over time. This sample would allow detecting a group difference of 14 percentage points (pp) for changes in EBF prevalence among children aged 0–5.9 mo [assuming baseline EBF of 55% (19)]. In addition, a total sample of 600 pregnant women (300/group) was sufficient to detect 15-pp difference in the women who consume ≥5 food groups/d.

#### Randomization.

A situational analysis of all of the subdistricts in 4 evaluation districts was conducted, assessing several factors associated with maternal and child health and nutrition. These factors included socioeconomic status, availability and use of health and nutrition services, educational level, access to water and sanitation, and participation in social welfare programs. Scores for these factors were summed for each subdistrict. Subdistricts with similar scores were matched and treated as a pair. Twenty subdistricts were randomly selected from these matched pairs through a manual lottery, then randomly assigned to either the nutrition-focused MNCH (10 subdistricts) or standard MNCH (10 subdistricts) group.

A household census was conducted at baseline and endline to create a list of pregnant women and mothers with infants <6 mo of age. Households were selected for surveys by using systematic sampling beginning with a random seed to yield the desired sample size per cluster. Women who could not understand and answer questions were excluded. The refusal rate was 0.9%; we replaced these women by randomly selecting others from the list.

#### Data collection.

Data were collected via face-to-face interviews with the use of a structured questionnaire that was prepared in English, translated into Bangla, then back-translated. Enumerators were trained by mixed methods (lecture, role-play, mock interview, and practice) in classroom and field settings.

Coverage and use of maternal nutrition interventions were assessed by asking recently delivered women about ANC (whether they received antenatal visits, the timing of the first visit, and the total number of visits), frontline worker visits (whether they were visited at home and how many times), and information received about nutrition for pregnant or lactating women and breastfeeding. Women were asked whether they received IFA and calcium supplements for free or purchased them, and if they participated in community mobilization activities.

The consumption of IFA and calcium supplements was assessed among recently delivered women by asking women to report how many IFA or calcium tablets they consumed during their last pregnancy. During monthly visits to women’s homes, health volunteers and workers observed empty strips and recorded the number of IFA and calcium tablets consumed in a mother-baby book (available for 88% of mothers); this book was used to assist women in their recall. The consumption of diversified foods and adequate amounts of macro- and micronutrients (excluding supplements) both inside and outside the home was assessed among pregnant women by using a 24-h diet recall, with the use of both standard and household unique recipes, with repeated measures in 20% of women to account for day-to-day variations. A Bangladeshi food-composition table (20) was used to calculate the nutrient content of foods for energy, macronutrients, and micronutrients. The probability of adequate intake was calculated as the probability that a woman’s intake is above the Estimated Average Requirement (EAR), adjusted for age and pregnancy (21). The 217 food items were also categorized into 10 food groups (22), with a minimum of 5 food groups/d as recommended for women of reproductive age to achieve their micronutrient needs (22).

Early breastfeeding practices were assessed among recently delivered women with infants aged <6 mo on the basis of the WHO-recommended indicators (23), including early initiation of breastfeeding (i.e., proportion of infants put to the breast within 1 h of birth), and EBF in the previous 24 h (i.e., proportion of infants aged 0–5.9 mo who were fed only breast milk). Other covariates possibly associated with the uptake of interventions were measured at the child (age and sex), mother (age, education, and occupation), and household (household size, number of children <5 y old, and socioeconomic status) levels. The measure of socioeconomic status was created from the first principal component (which explained 46% of the variance) by using variables on ownership of house and land, housing quality, access to services, and household assets (24, 25).

#### Statistical analysis.

Baseline differences between the 2 study groups were tested by using linear (continuous variables) or logit (categorical variables) regression models. Differential effects were tested by using regression models that estimated differences in changes over time between the 2 study groups (26), accounting for subdistricts as a random effect with the use of a cluster sandwich estimator (27). The best linear unbiased predictor of the individual’s usual intake was used to adjust for random within-person measurement error (28). Models for breastfeeding practices also adjusted for infant age and sex. By using a subset of 5 items adapted from Reynolds (29) that were collected in the endline survey, we tested for social desirability bias (i.e., tendency of respondents to respond so as to be viewed favorably by others) (30). Data analysis was performed with the use of Stata 13 (StataCorp).

#### Ethical approval.

Approval was obtained from the institutional review boards at the International Food Policy Research Institute and the Bangladesh Medical Research Council. All of the women were provided with detailed information in writing and verbally at recruitment. Written informed consent was obtained from all women ≥18 y old. For women <18 y of age, we obtained their assent and the permission of their guardians. The study was registered at clinicaltrials.gov as NCT02745249.

## Results

### 

#### Sample characteristics.

**FIGURE 1:**
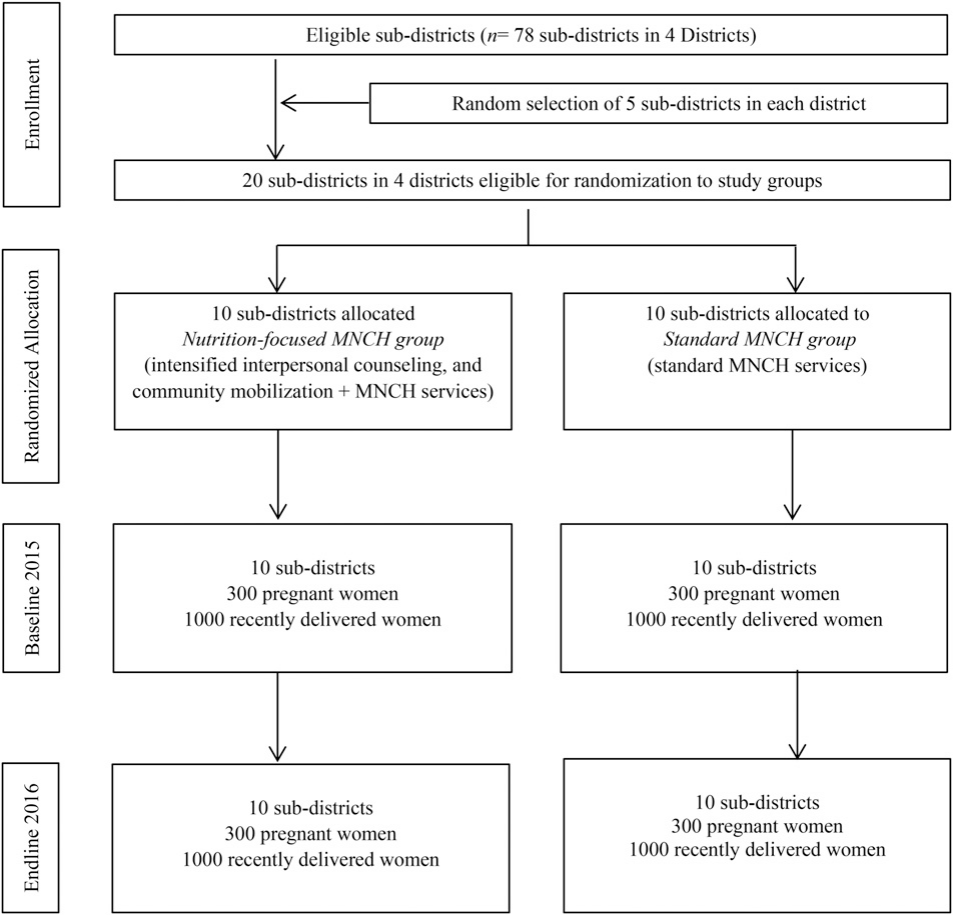
Trial profile. MNCH, Maternal, Neonatal, and Child Health.

No evaluation clusters were lost to follow-up, and none crossed from standard MNCH to nutrition-focused MNCH groups during implementation (**Figure 1**). The cluster size was similar across clusters and over time. The mean age of recently delivered women was 24 y (range: 13–44 y) (**Table 2**). The majority of women were housewives. On average, women had 6 y of education: >10% had no schooling and >80% did not complete high school. Randomization was successful with no differences in cluster, maternal, or household characteristics between study groups at baseline.

**TABLE 2:** Selected characteristics of pregnant and recently delivered women, by study group and survey round^1^

	Baseline		Endline	
	Standard MNCH	Nutrition-focused MNCH	Standard MNCH	Nutrition-focused MNCH
Pregnant women				
*n*	300	300	300	300
Gestational age, wk	6.2 ± 1.5^2^	6.2 ± 1.5	6.1 ± 1.5	6.2 ± 1.6
Second trimester, %	55.3	54.7	57.0	56.0
Third trimester, %	44.7	45.3	43.0	44.0
Age, y	23.7 ± 5.6	24.3 ± 5.6	24.0 ± 5.6	23.7 ± 5.6
Occupation as housewife, %	87.3	89.3	95.0	93.3
Education, %				
No schooling	12.7	11.7	9.7	9.3
Primary school	33.0	30.0	30.0	33.0
Secondary school	42.7	46.7	40.0	43.3
High school, college or higher	11.7	11.7	20.3	14.3
Muslim religion, %	93.3	93.3	93.0	94.0
Household characteristics				
Household size, *n*	4.1 ± 1.7	4.0 ± 1.7	4.2 ± 1.8	4.2 ± 1.6
Children <5 y of age, *n*	0.3 ± 0.5	0.3 ± 0.5	0.3 ± 0.5	0.3 ± 0.5
Socioeconomic index^3^	−0.05 ± 1.00	−0.16 ± 0.86	0.11 ± 1.06	0.11 ± 0.89
Recently delivered women				
*n*	1000	1000	1000	1000
Children’s age, mo	3.1 ± 1.7	3.1 ± 1.7	3.2 ± 1.7	3.2 ± 1.7
Maternal characteristics				
Age, y	24.2 ± 5.58	24.7 ± 5.43	25.1 ± 5.61	24.8 ± 5.40
Occupation as housewife, %	90.3	89.4	95.0	96.4
Education, %				
No schooling	12.8	10.7	12.0	9.00
Primary school	33.9	36.4	33.5	31.1
Secondary school	37.9	37.9	39.6	42.9
High school, college or higher	15.4	15.0	14.9	17.0
Muslim religion, %	93.5	93.6	92.7	93.9
Household characteristics				
Household size, *n*	5.0 ± 1.8	5.2 ± 1.9	5.0 ± 1.6	5.2 ± 1.9
Children <5 y of age, *n*	1.3 ± 0.5	1.3 ± 0.5	1.2 ± 0.4	1.3 ± 0.5
Socioeconomic index^3^	−0.06 ± 0.96	−0.06 ± 0.99	−0.03 ± 0.84	0.15 ± 0.98

1 Differences in groups at baseline and endline were tested by using ordinary least-squares regression models (continuous variables) or logit regression models (categorical variables), adjusting for clustering effect at the district and subdistrict levels; no difference was found. MNCH, Maternal, Neonatal, and Child Health.

2 Mean ± SD (all such values).

3 A socioeconomic index was constructed by using principal components analysis with variables on ownership and assets. It is a standardized score, with mean = 0 and SD = 1.

#### Effects on coverage and use of ANC and nutrition services.

Almost all of the women received ANC during pregnancy (**Table 3**). More women in the nutrition-focused MNCH group received early ANC visits (from the first trimester) than those in the standard MNCH group (effect: 18 pp). Household exposure to frontline workers was high in both groups (80–90%), but was significantly higher in nutrition-focused MNCH areas at endline. Mothers in the nutrition-focused MNCH group were visited more frequently than those in the standard MNCH group at endline by health workers [6.0 times (95% CI: 5.8, 6.1 times) compared with 3.7 times (95% CI: 3.6, 3.9 times)] and by health volunteers [8.1 times (95% CI: 7.8, 8.5 times) compared with 3.2 times (95% CI: 2.9, 3.4 times)].

**TABLE 3:** Coverage and use of ANC services and nutrition interventions among recently delivered women^1^

	Baseline^2^		Endline		
	Standard MNCH	Nutrition-focused MNCH	Standard MNCH	Nutrition-focused MNCH	Difference-in-difference effect estimates^3^
*n*	1000	1000	1000	1000	
Received ANC and contacts with frontline workers					
Received any ANC	97.5	98.4	98.2	99.0	−0.1
Received ANC from first trimester	46.5	45.3	47.3	63.9	17.8^∗^
Received ANC ≥4 times	78.7^∗∗^	84.2	81.9	90.6	5.2
Had ever been visited at home by health worker	91.2^∗∗∗^	96.2	88.2	97.3	4.1
Number of times visited by health worker	2.4 ± 2.1^4,5^	2.4 ± 1.9	3.7 ± 2.4	6.0 ± 2.6	2.27^∗∗^
Had ever been visited at home by health volunteer	83.1^∗∗^	87.9	69.6	93.1	18.7^∗∗∗^
Number of times visited by health volunteer	3.1 ± 3.4^6^	3.0 ± 2.9	3.2 ± 3.4	8.1 ± 5.3	5.12^∗∗^
Received any information about nutrition for pregnant/lactating women	94.5^∗∗∗^	98.2	96.5	98.9	−1.3
Eat 5 varieties of foods	36.6^∗∗^	29.5	22.9	82.3	66.5^∗∗∗^
Eat additional amounts of food	61.2	62.0	76.2	69.4	−7.60
Measuring weight	19.2	17.7	23.1	60.1	38.5^∗∗^
Weight gain	12.2	13.6	12.9	28.2	13.9
Taking IFA	45.5^∗∗∗^	55.1	66.9	87.1	10.6
Taking calcium	44.1^∗∗∗^	53.3	66.1	87.9	12.6
Received any messages about breastfeeding	86.3	87.4	89.1	97.8	7.6
Initiate breastfeeding within the first hour of birth	65.2	67.6	63.6	78.7	12.7
Feed colostrum	48.5	51.2	66.8	76.0	6.5
Do not put anything in child’s mouth after birth	31.8	29.5	34.8	48.5	16.0^∗^
Feed only breast milk to child for 6 mo after birth	43.6	42.7	51.2	57.6	7.30
Feed expressed breast milk	5.9^∗∗^	3.0	4.2	9.9	8.6^∗∗^
Received only free IFA during pregnancy	53.3	44.8	42.4	96.5	62.6^∗∗∗^
Purchased IFA during pregnancy	36.5	45.0	45.2	2.1	−52.8^∗∗∗^
Received only free calcium during pregnancy	42.4^∗^	31.5	34.9	96.3	72.3^∗∗∗^
Purchased calcium during pregnancy	44.1^∗∗^	55.0	51.0	2.3	−59.5^∗∗∗^
Weighed during last pregnancy	63.7	60.6	60.3	98.2	41.0^∗∗∗^
Number of times weighed	2.88 ± 2.1	2.9 ± 1.83	2.8 ± 1.6	6.1 ± 2.2	3.34^∗∗∗^

1 ^∗^*P* < 0.05, ^∗∗^*P* < 0.01, ^∗∗∗^*P* < 0.001. ANC, antenatal care; IFA, iron and folic acid; MNCH, Maternal, Neonatal, and Child Health.

2 Differences in groups at baseline were tested by using ordinary least-squares regression models (continuous variables) or logit regression models (categorical variables), adjusting for clustering effect at the district and subdistrict levels.

3 Difference-in-difference effect estimates between baseline and endline adjusted for clustering effect at the district and subdistrict levels.

4 Mean ± SD (all such values).

5 Ranges from 0 to 10 visits at baseline and 0 to 16 visits at endline.

6 Ranges from 0 to 16 visits at baseline and 0 to 24 visits at endline.

More than ninety percent of recently delivered women reported having received information about nutrition for pregnant and lactating women. Mothers in the nutrition-focused MNCH group reported a significantly higher exposure to messages on nutrition during pregnancy (effect: 66.5 pp for eating a variety of foods and 38.5 pp for measuring weight) and on breastfeeding practices (effect: 16 pp for not feeding the child anything other than breast milk after birth and 8.6 pp for feeding expressed breast milk). At endline, most women in nutrition-focused MNCH areas received free IFA (96.5%) and calcium (96.3%) tablets, mainly from BRAC staff. The percentage of women who were weighed and the number of times they were weighed during pregnancy were higher for women in nutrition-focused MNCH areas than for those in standard MNCH areas. In nutrition-focused MNCH areas, ∼40–50% of all mothers reported ever having seen video shows in the community, which was significantly higher than in standard MNCH areas.

#### Effects on reported consumption of IFA and calcium supplements.

**FIGURE 2:**
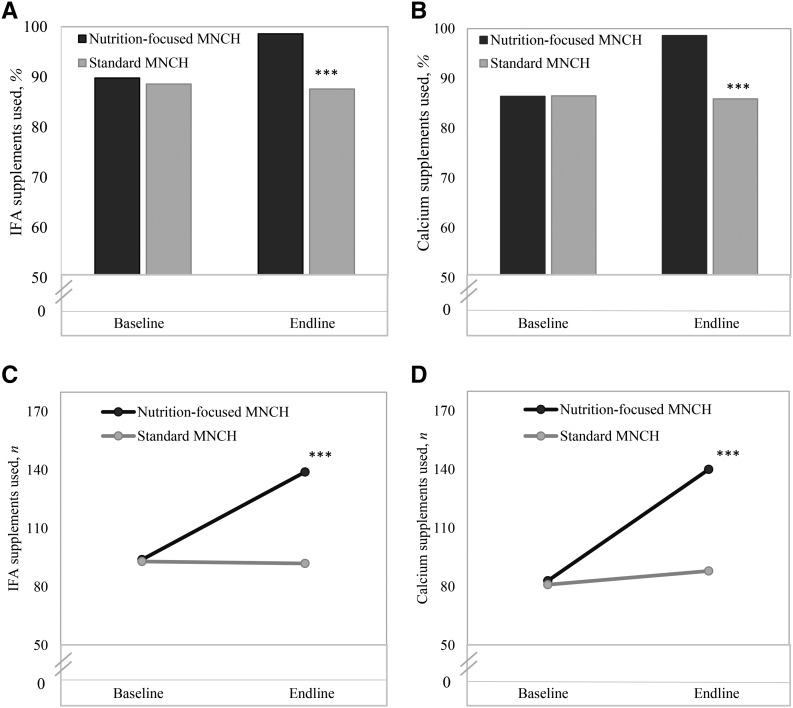
Consumption of IFA and calcium supplements in recently delivered women, by program group and survey round, for percentages of women who ever used IFA (A) or calcium (B) supplements and numbers of women who used IFA (C) or calcium (D) supplements. The recommended dosage for pregnancy was 180 tablets of IFA and calcium. Difference-in-difference effect estimates between baseline and endline adjusted for clustering effect at the district and subdistrict levels. ∗∗∗*P* < 0.001. IFA, iron and folic acid; MNCH, Maternal, Neonatal, and Child Health.

The reported consumption of IFA and calcium supplements was high in both groups at baseline but improved substantially more over time in the nutrition-focused MNCH areas than in the standard MNCH areas (**Figure 2**). Effect estimates for having consumed IFA and calcium supplements during pregnancy were 9.8 and 12.8 pp, respectively. Significant effects were also seen for the number of IFA and calcium tablets consumed (effects: 46 and 50 tablets, respectively).

#### Effects on consumption of diversified foods and adequate amounts of macro- and micronutrients.

Significant effects were observed for the number of food groups consumed (effect: 1.6 food groups) and the proportion of women who consumed ≥5 food groups/d (effect: 30.0 pp) (**Table 4**). Effects were also seen for individual food group consumption, both in the proportion who consumed a variety of foods and in the amounts consumed. Compared with the standard MNCH group, the nutrition-focused MNCH group showed higher increases in the proportion of women who consumed pulses, dairy, meat, eggs, and vitamin A–rich fruit and vegetables (effect range: 17–38 pp). For quantity consumed, increases were greater in the nutrition-focused MNCH group than in the standard MNCH group for most food groups, except for staples and other fruit. Average per capita energy intake (∼2300 kcal/d) increased in both groups with a nondifferential impact. The nutrition-focused MNCH program had significant effects on daily intakes of protein and fats (effects: 27 and 34 g, respectively) and on most micronutrients studied (effects: 356 mg for calcium, 6.4 mg for iron, 3.3 mg for zinc, 156 g for vitamin C, 291 μg for folate, 1.7 μg for vitamin B-12 and 706 μg for vitamin A) (**Table 5**). The probability of an adequate intake (above the EAR) increased more in the nutrition-focused MNCH group than in the standard MNCH group at endline, except for intakes of niacin, vitamin C, and vitamin B-6. Effect estimates for other micronutrients ranged from 5 to 37.2 pp.

**TABLE 4:** Diversity and quantity of food groups consumed during past 24 h by pregnant women, by program group and survey round^1^

	Baseline^2^		Endline		
	Standard MNCH	Nutrition-focused MNCH	Standard MNCH	Nutrition-focused MNCH	Difference-in-difference effect estimates^3^
*n*	300	300	300	300	
Type of food groups consumed, %					
All starchy staple foods	100	100	100	100	—
Pulses	37.7	34.7	37.0	65.7	29.3^∗∗∗^
Nuts and seeds	3.7	1.6	3.7	3.0	1.4
Dairy	40.3	34.3	39.3	67.0	33.7^∗∗^
Flesh foods	82.7	85.7	90.3	93.0	−0.30
Meat	23.0	20.7	16.0	30.7	17.0^∗∗^
Fish	73.0	79.0	85.3	84.3	−6.70
Eggs	26.7	24.3	25.7	61.3	38.0^∗∗∗^
Dark-green leafy vegetables	44.7	49.7	39.0	67.3	23.3^∗^
Other vitamin A–rich fruit and vegetables	28.0^∗^	20.7	17.0	46.0	36.0^∗∗∗^
Other vegetables	90.0	93.0	98.3	93.7	−7.70^∗^
Other fruit	62.7^∗^	52.7	58.7	53.3	4.70
Number of food groups consumed, *n*	5.1 ± 1.4^4^	5.0 ± 1.4	5.1 ± 1.3	6.5 ± 1.6	1.6^∗∗∗^
Consumed ≥5 food groups, %	67.0	60.7	65.0	88.7	30.0^∗∗∗^
Quantity of food groups consumed, g					
All starchy staple foods	574 ± 210	574 ± 225	637 ± 399	611 ± 276	−26.3
Pulses	89.9 ± 79.7	87.9 ± 77.8	121 ± 96.9	208 ± 121	88.7^∗∗∗^
Nuts and seeds	1.9 ± 10.9	3.8 ± 20.2	3.8 ± 14.6	2.6 ± 12.1	−3.0
Dairy	93.0 ± 171	77.8 ± 141	93.2 ± 137	171 ± 155	93.1^∗∗^
Flesh foods	89.6 ± 92.0	83.0 ± 91.7	116 ± 120	196 ± 186	87.4^∗∗^
Meat	22.2 ± 61.4	21.0 ± 57.7	23.1 ± 63.3	49.1 ± 114	27.2^∗∗^
Fish	67.4 ± 77.5	62.4 ± 73.1	97.9 ± 111	151 ± 151	58.1^∗^
Eggs	14.3 ± 32.1	12.9 ± 25.6	19.1 ± 36.3	37.7 ± 37.7	20.1^∗∗^
Dark-green leafy vegetables	127 ± 185	138 ± 174	123 ± 145	334 ± 272	200^∗∗∗^
Other vitamin A–rich fruit and vegetables	36.3 ± 97.4	33.3 ± 91.8	26.6 ± 92.8	91.7 ± 174	68.0^∗∗^
Other vegetables	139 ± 111	128 ± 112	167 ± 129	212 ± 141	56.1^∗∗^
Other fruit	185 ± 233^∗∗^	131 ± 272	141 ± 197	160 ± 201	72.4

1 ^∗^*P* < 0.05, ^∗∗^*P* < 0.01, ^∗∗∗^*P* < 0.001. MNCH, Maternal, Neonatal, and Child Health.

2 Differences in groups at baseline were tested by using ordinary least-squares regression models (continuous variables) or logit regression models (categorical variables), adjusting for clustering effect at the district and subdistrict levels.

3 Difference-in-difference effect estimates between baseline and endline adjusted for clustering effect at the district and subdistrict levels.

4 Mean ± SD (all such values).

**TABLE 5:** Average per capita vitamin and mineral intakes of pregnant women, by program group and survey round^1^

			Baseline^2^		Endline		
	EAR	RDA	Standard MNCH	Nutrition-focused MNCH	Standard MNCH	Nutrition-focused MNCH	Difference-in-difference effect estimates^3^
*n*			300	300	300	300	
Average intake							
Energy, kcal/d	—	—	2354 ± 778^4^	2310 ± 864	2536 ± 1339	2931 ± 1100	437
Protein, g/d	—	—	67.2 ± 26.4	65.7 ± 26.9	72.1 ± 42.8	97.8 ± 46.8	27.2^∗^
Fat, g/d	—	—	40.6 ± 33.1	36.5 ± 25.9	48.5 ± 36.1	78.5 ± 54.1	34.1^∗^
Carbohydrate, g/d	—	—	447 ± 149	448 ± 171	473 ± 255	509 ± 217	36.0
Calcium, mg	—	1000	381 ± 369	374 ± 386	357 ± 340	705 ± 480	356^∗∗∗^
Iron, mg	22	27	10.3 ± 6.13	10.2 ± 5.60	9.88 ± 6.76	16.2 ± 9.43	6.41^∗∗^
Zinc, mg	9.5	11	7.75 ± 3.14	7.53 ± 3.13	8.01 ± 4.68	11.1 ± 5.10	3.28^∗^
Vitamin C, mg	70	85	308 ± 336^∗∗^	231 ± 264	264 ± 326	343 ± 347	156^∗∗^
Thiamin, mg	1.2	1.4	1.66 ± 0.61	1.65 ± 0.66	1.77 ± 1.09	2.35 ± 1.12	0.59
Riboflavin, mg	1.2	1.4	0.96 ± 0.69	0.95 ± 0.67	0.96 ± 0.74	1.65 ± 1.05	0.70^∗∗^
Niacin, mg	14	18	29.5 ± 9.8	29.9 ± 11.4	31.9 ± 17.8	35.8 ± 15.8	3.61
Vitamin B-6, mg	1.6	1.9	3.08 ± 1.08	3.09 ± 1.15	3.25 ± 1.85	3.89 ± 1.73	0.62
Folate (total), μg	520	600	309 ± 266	287 ± 234	283 ± 290	552 ± 395	291^∗∗^
Vitamin B-12, μg	2.2	2.6	1.53 ± 1.47	1.47 ± 1.58	2.12 ± 3.07	3.73 ± 4.75	1.65^∗∗^
Vitamin A (RAE), μg	550	770	410 ± 585	405 ± 708	357 ± 619	1058 ± 1157	706^∗∗∗^
Probability of adequate intake, %							
Calcium	—	—	37.8	25.3	20.3	45.0	37.2^∗∗∗^
Iron	—	—	0.3	0.1	1.4	6.0	4.8^∗^
Zinc	—	—	27.7	25.3	30.1	52.1	24.5^∗∗^
Vitamin C	—	—	73.5	72.5	52.3	64.7	13.4^∗∗^
Thiamin	—	—	62.5	62.6	56.3	69.7	13.3^∗∗^
Riboflavin	—	—	14.2	14.9	19.3	45.6	25.6^∗∗∗^
Niacin	—	—	74.9	75.0	73.2	74.5	1.2
Vitamin B-6	—	—	74.9	75.0	71.5	74.6	3.0
Folate (total)	—	—	6.3	4.6	6.2	26.8	22.3^∗∗∗^
Vitamin B-12	—	—	15.6	11.9	24.1	42.7	22.3^∗∗^
Vitamin A (RAE)	—	—	11.1	9.8	10.8	38.2	28.8^∗∗∗^

1 EARs and RDAs are based on the Institute of Medicine’s recommendation for pregnant women. ^∗^*P* < 0.05, ^∗∗^*P* < 0.01, ^∗∗∗^*P* < 0.001. EAR, Estimated Average Requirement; MNCH, Maternal, Neonatal, and Child Health; RAE, retinol activity equivalent.

2 Differences in groups at baseline were tested by using ordinary least-squares regression models (continuous variables) or logit regression models (categorical variables), adjusting for clustering effect at the district and subdistrict levels.

3 Difference-in-difference effect estimates between baseline and endline adjusted for clustering effect at the district and subdistrict levels.

4 Mean ± SD (all such values).

#### Effects on breastfeeding practices.

**FIGURE 3:**
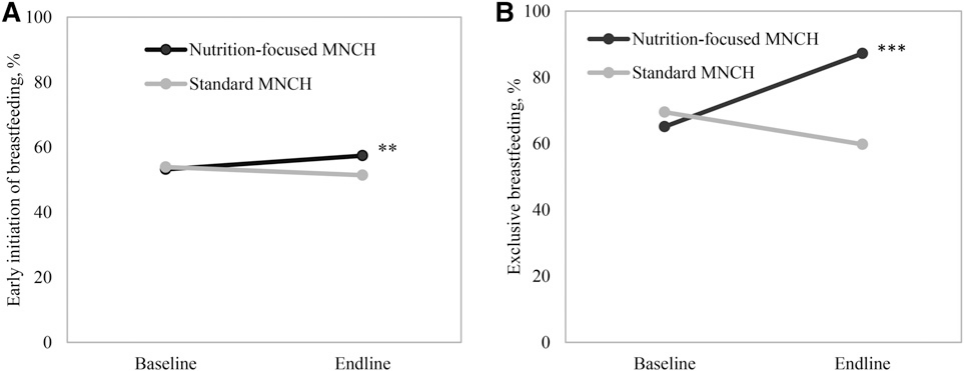
Breastfeeding practices, by program group and survey round, for early initiation of breastfeeding (A) and exclusive breastfeeding (B). Difference-in-difference effect estimates between baseline and endline adjusted for clustering effect at the district and subdistrict levels. **P < 0.01; ***P < 0.001. MNCH, Maternal, Neonatal, and Child Health.

The nutrition-focused MNCH program had a large, significant effect on the proportion of mothers who reported exclusively breastfeeding their infants 0–6 mo of age (effect: 31 pp), reaching 87% at endline (**Figure 3**). Early initiation of breastfeeding slightly increased in nutrition-focused MNCH areas, but no significant differential effect was observed.

#### Accounting for potential social desirability bias in main outcome measures.

We found no evidence of a social desirability bias for breastfeeding practices and number of food groups consumed, but there was a tendency for women with higher social desirability scores to report higher consumption of IFA tablets in both groups and higher consumption of calcium tablets and greater dietary diversity in the standard MNCH group (**Supplemental Text 1**, **Supplemental Table 1**). The observed differential effects of the intervention groups on IFA, calcium, and dietary consumption were slightly attenuated after adjusting for social desirability scores, but remained significant (**Supplemental Table 2**).

## Discussion

Intensified interpersonal counseling and community mobilization to address nutrition during pregnancy, delivered through an existing MNCH program, significantly improved multiple outcomes such as coverage and use of maternal nutrition interventions, maternal dietary diversity, micronutrient supplement consumption, and EBF practices. Given the short 1-y period of intervention, the effects were substantial, showing an increase in women who consumed IFA and calcium tablets by 46 and 50 pp, respectively; a 30-pp increase in the women who consumed ≥5 food groups/d; an increase in individual food groups consumed ranging from 16 to 36 pp; a significant increase in intakes of most micronutrients studied; and a 31-pp increase in the women exclusively breastfeeding their infant 0–6 mo of age in the nutrition-focused MNCH compared with the standard MNCH group.

The effects of the interventions on maternal IFA consumption and dietary diversity are consistent with previous literature on IFA distribution through ANC (31) and nutrition education counseling (5, 6). The effect on EBF was similar to results from systematic reviews of breastfeeding-promotion interventions (32, 33) and comparable to previous studies in Bangladesh and Vietnam (30). In contrast with previous studies that mostly reported on findings from single interventions, our study provides evidence of effects from a comprehensive integrated package of maternal health and nutrition interventions that map to the new WHO ANC guidelines (3).

The effects of the nutrition-focused MNCH program were likely due to a well-designed and locally relevant package of maternal nutrition interventions, high quality of service delivery, and high service coverage of the base MNCH program. The strategic use of data from different sources (program conceptual framework, formative research, and evidence from other existing studies) to carefully design the context-specific package of proven nutrition interventions (34) resulted in a program that could be effectively delivered under real-life conditions as part of the MNCH program. The program placed priority on service delivery, including ensuring adequate supplies, high-quality training, close supervision, well-defined roles and job aids for frontline workers, and performance-based incentives. In a previous behavior-change program in Bangladesh, an incentive package played an important role in motivating frontline workers, particularly the health volunteers who are not paid, to join and retain their jobs (35, 36), be willing to spend extra time required, and comply with the program protocol (37). Interventions were also designed to engage husbands, mothers-in-law, and the broader community to support mothers in obtaining foods and other supplies needed to achieve the recommended practices. Coverage of intervention was high. At endline, in the nutrition-focused MNCH areas, nearly all of the women reported having received the various intensified nutrition interventions, such as information about maternal nutrition, free IFA and calcium tablets, and weight measurement during pregnancy.

Rapid integration and high coverage of interventions were facilitated by the strong health delivery system already in place. BRAC’s MNCH program presented a solid foundation for this study; its MNCH program (38) has been operating at a large scale in both rural (14 districts, reaching 25 million population) and urban (11 city corporations and 2 municipalities, reaching 7 million populations) areas since 2008 (39), and its services are well received by the communities it serves (40). BRAC’s networks of health volunteers and workers are motivated and well supervised, and cover small catchment areas, making it a health program with a high potential for success. Furthermore, our results show that the BRAC health volunteers and workers were capable of incorporating new skills and tasks in their work, such as counseling to promote behavior change, measuring women’s weight gain during pregnancy, and conducting more frequent home visits.

We found no effect on early initiation of breastfeeding, a practice adopted by only half of the mothers in both groups, whereas large effects on EBF were achieved. This may be due to several factors related to childbirth and delivery. Cesarean delivery, which is negatively associated with early initiation of breastfeeding (41), increased from 22% to 25% between baseline and endline in both the intervention and control areas. Approximately 60% of mothers gave birth at home where they received advice from traditional birth attendants, mothers-in-law, and other relatives to promote traditional feeding practices such as prelacteal feeding and discarding colostrum; these practices are barriers to the early initiation of breastfeeding in Bangladesh and other South Asian countries (42). Tailored messages and additional support around delivery, either at home or in health facilities, are needed to improve early initiation of breastfeeding in these contexts.

Despite effects on most micronutrients studied, the probability of adequate intakes of micronutrients from food remained low. In the nutrition-focused MNCH group, the probability of adequate intakes from food was 6% for iron, 27% for folate, and 45% for calcium. This reinforces the importance of combining both nutrition counseling and micronutrient supplementation. Given that the supplements are designed to support healthy pregnancy, and that their concentration is higher or close to the EARs for iron and folic acid, respectively, and half the Adequate Intake for calcium, regular intakes of the supplements likely lead to a high probability of adequate intake for most women in the intervention group where nearly all of them (98%) consumed supplements.

Our self-reported measures for intake of IFA, calcium, and dietary diversity may have had a social desirability bias, because these behaviors are recommended as part of the MNCH program. For IFA and calcium, we aided recall by referring to the mother-baby books where available. The results for breastfeeding practices were unaffected by respondents’ desire for social approval, and the impacts on the consumption of IFA and calcium supplements remained strongly significant even after accounting for social desirability bias. Our routine monitoring data showed similar results of increasing IFA and calcium consumption in the intervention group, which support the results from evaluation study. In terms of generalizability, this study was carried out in the context of a well-functioning and robust MNCH program; implementing a similar program in another program that functions less well likely would result in smaller effects.

This study showed the feasibility and effects of integrating nutrition interventions into the existing MNCH program even within a short 1-y period. Lessons learned from this intervention, together with training manuals, job aids, and other materials, are being shared with the Bangladesh government and elsewhere in the efforts to integrate and reinforce nutrition interventions delivered through routine health care services. Future studies should include objective measures, such as biomarkers for iron and calcium, and examine whether successful integration of nutrition interventions into an existing high-quality MNCH large-scale program leads to nutrition effects beyond improved maternal diets and micronutrient intakes and improved longer-term newborn, infant, and child health outcomes.

In conclusion, MNCH programs can provide essential health and nutrition interventions to improve maternal and child nutrition, health, and survival. This innovative study showed that the integration of nutrition interventions into ANC is effective when implemented well, particularly when facilitated by a solid and functional system for early pregnancy detection and ANC service delivery. This effective model could be adapted into other health systems in similar contexts, particularly for program models that rely on incentivized frontline workers for conducting home-based interpersonal counseling and community mobilization (43, 44). To ensure successful implementation and impact, special consideration is also needed with regard to strengthening existing health services and a functional network of skilled frontline workers.
